# Clinical Characteristics and Outcomes of Pediatric COVID-19 Pneumonia Treated with Favipiravir in a Tertiary Care Center

**DOI:** 10.3390/v16060946

**Published:** 2024-06-12

**Authors:** Phanthila Sitthikarnkha, Rawisara Phunyaissaraporn, Sirapoom Niamsanit, Leelawadee Techasatian, Suchaorn Saengnipanthkul, Rattapon Uppala

**Affiliations:** Department of Pediatrics, Faculty of Medicine, Khon Kaen University, 123 Mittraphap Road, Muang, Khon Kaen 40002, Thailand; puntsi@kku.ac.th (P.S.); rawiph@kku.ac.th (R.P.); sirani@kku.ac.th (S.N.); leelawadee@kku.ac.th (L.T.); suchsa@kku.ac.th (S.S.)

**Keywords:** COVID-19, monocytosis, pneumonia, children, favipiravir

## Abstract

The COVID-19 pandemic, caused by SARS-CoV-2, has posed significant health challenges worldwide. While children generally experience less severe illness compared to adults, pneumonia remains a substantial risk, particularly for those under five years old. This study examines the clinical characteristics and treatment outcomes of pediatric COVID-19 pneumonia patients treated with favipiravir in Thailand, aiming to identify associated factors for pneumonia. A retrospective review was performed on pediatric patients aged 1 month to 18 years hospitalized with COVID-19 at Srinagarind Hospital, Khon Kaen University, from 13 January 2020 to 15 November 2021. Data on demographics, clinical symptoms, treatment, and outcomes were collected, and logistic regression analysis was used to identify factors associated with pneumonia. Among 349 hospitalized children, the median age was 8 years, with 51.9% being male. Symptoms included a fever (100%), a cough (74.2%), and a rash (24.9%). COVID-19 pneumonia was diagnosed in 54.7% of the children. Favipiravir was administered as the standard treatment, showing mild adverse effects, including a rash (4.3%) and nausea (2.8%). Monocytosis was significantly associated with COVID-19 pneumonia (aOR 30.85, 95% CI: 9.03–105.41, *p* < 0.001), with an ROC curve area of 0.77 (95% CI: 0.71–0.83). Pediatric COVID-19 patients typically exhibit mild-to-moderate symptoms, with pneumonia being common in the early pandemic phase. Monocytosis is a significant factor associated with COVID-19 pneumonia. Favipiravir demonstrated mild adverse effects. Further studies are needed to validate these findings across different settings and phases of the pandemic.

## 1. Introduction

The global outbreak of coronavirus, an infectious disease caused by severe acute respiratory syndrome coronavirus 2 (SARS-CoV-2), is referred to as the coronavirus disease 2019 (COVID-19). The outbreak of COVID-19 was officially classified as a pandemic by the World Health Organization on 11 March 2020 [1]. Globally, the COVID-19 pandemic has resulted in six million fatalities [2]. The COVID-19 infection was initially identified in Thailand in January 2020, and the disease subsequently continued to propagate [3]. Most COVID-19 patients are routinely hospitalized in Thailand in the initial stages of its spreading [4]. Despite the fact that the severity of COVID-19 in children is comparatively lower than that observed in adults, pneumonia remains a substantial national concern, particularly among children aged below five years, due to its status as the primary cause of mortality among young patients [2].

A nationwide study conducted in Colombia disclosed significant insights into pediatric COVID-19 cases, with 9.2% of the total confirmed cases affecting children under the age of 18. This research underscored that, despite the relatively low mortality rate among pediatric patients, younger children, particularly those under the age of six, were more susceptible to severe clinical conditions [5]. In Turkey, a study was conducted to predict lung involvement using laboratory and clinical results in 101 pediatric COVID-19 patients. A fever and a cough were prevalent, and 32.7% of patients exhibited lung involvement during CT imaging. The other laboratory data did not differ between the groups, despite the fact that fibrinogen levels were significantly correlated with pulmonary involvement [6].

In Thailand, like many other regions, the early phases of the pandemic saw routine hospitalization of COVID-19 patients to curb the spread and manage complications. The clinical management of pediatric COVID-19 cases in Thailand often involves the use of favipiravir as per national treatment guidelines. Favipiravir is the treatment of choice according to national clinical practice guidelines when a patient needs hospitalization, with a dosage of 70 mg/kg/day twice daily on the first day, followed by 30 mg/kg/day twice daily on days 2–5 [7]. Limited data exist regarding the efficacy and adverse effects of favipiravir in pediatric patients, which hinders its substantial support.

Comprehensive data on the efficacy and safety of favipiravir specifically in pediatric populations remain limited. Consequently, the objective of this research endeavor is to examine the clinical attributes and results of favipiravir-treated pediatric COVID-19 pneumonia, in addition to identifying factors associated with COVID-19 pneumonia. This research provides essential insights that could optimize pediatric COVID-19 management, reducing the incidence of severe outcomes, and improving overall healthcare practices. Through a thorough retrospective review, this study endeavors to enhance our understanding of favipiravir’s role in pediatric COVID-19 treatment and lay the groundwork for future research to corroborate these findings in diverse settings and the evolutionary phases of the pandemic.

## 2. Materials and Methods

### 2.1. Study Design and Participants

We conducted a retrospective review of pediatric patients aged 1 month to 18 years who tested positive for COVID-19 through reverse transcription–polymerase chain reaction (RT-PCR) from 13 January 2020 to 15 November 2021, based on the official electronic medical record (Health Object^®^ program), at the Srinagarind Hospital, Faculty of Medicine, Khon Kaen University, Thailand. All confirmed COVID-19 patients were required to be hospitalized for isolation during the study period. Patients with multisystem inflammatory syndrome in children (MIS-C) and perinatal transmission were excluded from the study.

### 2.2. Data Collections

We collected data on the patients’ age, gender, underlying conditions, symptoms at admission, and treatment. Complete blood count results were obtained from all children hospitalized with COVID-19 infection. The outcomes of COVID-19 infection contained the number of respiratory failures that required endotracheal intubation or non-invasive ventilation such as a high-flow nasal cannula, the length of stay (LOS), if a pediatric intensive care unit was needed, and mortality.

**Figure 1:**
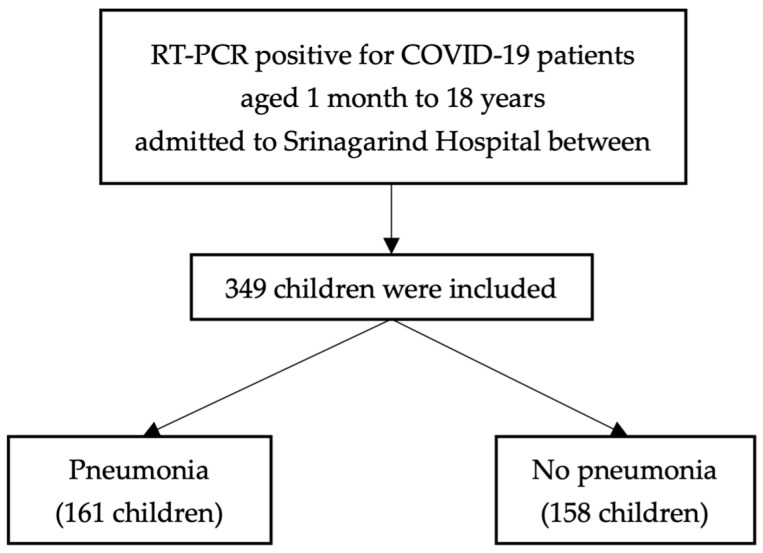
Flow diagram of the study.

In order to assess factors associated with COVID-19 pneumonia, we divided the patients into two groups: those with pneumonia and those without (Figure 1). Pneumonia was identified when a patient had an abnormal chest radiograph. All chest radiographs of pediatric COVID-19 patients were meticulously reviewed by radiologists and/or pediatric pulmonologists to ensure accuracy.

### 2.3. Ethical Consideration

This study was approved by the institutional review board of Khon Kaen University (#HE641626). Informed consent was waived since the study was retrospective in nature.

### 2.4. Statistical Analyses

Categorical variables were presented as frequencies and percentages. Upon data distribution, continuous variables were summarized as medians with interquartile ranges (IQRs). A univariate logistic regression analysis was applied to determine independent factors associated with COVID-19 pneumonia and presented as a crude odds ratio (OR) with a 95% confidence interval (95% CI). Backward stepwise multivariable logistic regression analysis was then performed. The selection of independent factors for the multivariable model was based on statistical significance in the univariable analysis (*p* < 0.05) and presented as an adjusted odds ratio (aOR). The goodness of fit of the model was assessed using the area under the receiver operating characteristic (ROC) curve to assess the predictive factor’s ability to discriminate COVID-19 pneumonia. Statistical significance was set at *p* < 0.05. All statistical analyses were conducted using Stata software version 10 (StataCorp LP, College Station, TX, USA).

## 3. Results

During the study period, there were 349 children hospitalized with COVID-19 infections. The median age of them was 8 years (IQR 3–14). Of the hospitalized children, 181 (51.86%) were boys. Among the cases, 41 had underlying diseases, with chronic lung disease (30 cases, 8.59%) being the most prevalent. The hospitalized children with COVID-19 exhibited symptoms such as a fever, a cough, a rash, and diarrhea. All the children had a fever; a cough was the second most common symptom, accounting for 259 cases (74.21%), followed by a rash (87 cases, 24.9%) and diarrhea (18 cases, 52%). There were 199 (54.73%) children diagnosed with COVID-19 pneumonia. (Table 1)

The laboratory results included a complete blood count and C-reactive protein (CRP). The median C-reactive protein was 1 mg/L, with an IQR of 0.49–4.59. The median white cell count and platelet count were 7940 (IQR 5630–9900) and 291,000 (247,000–363,000) cells/µL, respectively. Monocytosis, which is defined as an absolute monocyte greater than 800 cells/µL, was found in 88 children with COVID-19 infection (Table 2).

All the patients received favipiravir as standard treatment for the COVID-19 infection. In the study population, the adverse effects of favipiravir included a rash in 15 cases (4.30%), nausea and vomiting in 10 cases (2.87%), and bluish corneal discoloration in 2 cases. Endotracheal intubation was performed in four cases (1.15%), with three of them having chronic lung diseases and one having congenital cyanotic heart disease. Additionally, a heated humidified high-flow nasal cannula (HHHFNC) was used in seven cases (2.01%). The median length of stay was 12 days (IQR 10–13). Unfortunately, one patient died from underlying congenital cyanotic heart disease, but 348 cases showed improvement.

**Figure 2:**
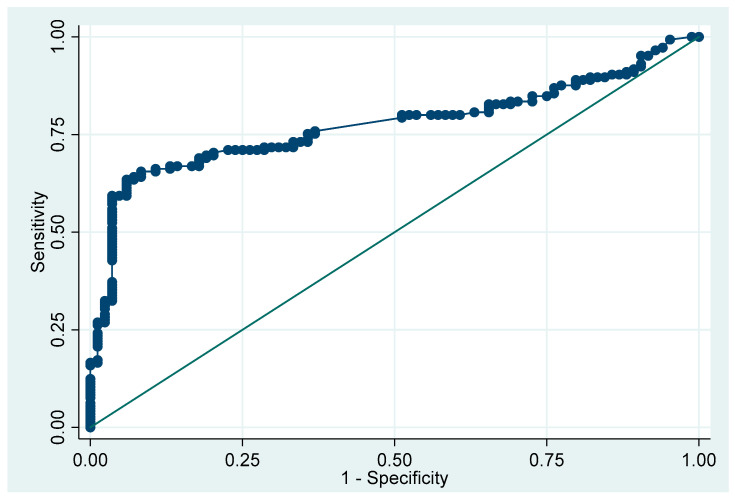
The area under the ROC curve for monocytosis in predicting pneumonia in pediatric patients with COVID-19 infections.

The analysis revealed that the CRP and monocyte levels were potentially associated with COVID-19 pneumonia (Table 3). After conducting a multivariate analysis, we found that monocytosis was the only factor associated with COVID-19 pneumonia in the children (aOR 30.85, 95% CI: 9.03 to 105.41, *p* < 0.001), while CRP did not show statistical significance (aOR 1.07, 95% CI: 0.96 to 1.19, *p* = 0.242). The area under the ROC curve for monocytosis in predicting COVID-19 pneumonia was 0.77 (95% CI: 0.71 to 0.83) (Figure 2).

## 4. Discussion

COVID-19 remains a substantial concern in the field of public health, characterized by unforeseen outbreaks and widespread distribution across the globe. This is particularly evident in children due to the highly contagious nature of the disease and its propensity to spread via respiratory particles. Institutional settings, including playgroups, schools, and daycares, pose an elevated hazard of virus transmission to children [8].

Wong et al. conducted an observational study that aligns with our findings that a significant proportion of the COVID-19 patients exhibited a fever (100%), a cough (74.21%), and moderate symptoms [1]. Pneumonia was observed in 32.6 percent of COVID-19 infections in the study by Satdhabudha et al., but only 54.7 percent in our investigation [2]. Due to the fact that many of our patients in Khon Kaen were gravely ill and due to the initial phase of COVID-19 spreading, most symptomatic patients needed hospitalization. As a result, a comparatively elevated incidence of pneumonia was documented in contrast to prior investigations. Low severity characterizes COVID-19 infections; our study’s mortality rate was 0.3 percent, which is consistent with the findings of Badal et al., who conducted a meta-analysis and reported a mortality rate of 0.3% [9].

The laboratory results were not reported in the preponderance of the studies. High CRP levels were associated with pneumonia according to the laboratory data collected; however, they were not statistically significant when confounding factors were controlled, which is consistent with the findings of Ozger HS et al. [10]. Monocytosis was identified as an independent risk factor for pediatric COVID-19 pneumonia in our study. Monocytosis, as indicated by the CBC, is correlated with a diagnosis of COVID-19 [11]. To the best of our knowledge, this is the first study to investigate monocytosis as a contributing factor to COVID-19 pneumonia in children. Previous studies have established significant associations between monocytes and COVID-19 severity. For instance, monocytes and macrophages have been implicated in the hyperactivation and overproduction of pro-inflammatory cytokines in severe COVID-19 cases, leading to cytokine storms and severe outcomes, including acute respiratory distress syndrome (ARDS) [12]. In addition, monocytes are recognized for their ability to infiltrate the lungs, thereby contributing to tissue damage and fibrosis. Two novel severe-disease-specific monocyte subsets, Mono 0 and Mono 5, were identified, and they exhibited pro-fibrogenic and pro-inflammatory characteristics. These subsets were not only present in patients with severe disease, but they were also identifiable in moderately ill patients who subsequently developed severe disease, indicating their potential as early markers for severe COVID-19 [13].

Our findings align with previous reports that underscore the critical role of monocytes in COVID-19 pathogenesis and highlight the importance of targeting these cells to mitigate disease severity. In patients with severe viral infections, certain cytokine profiles could function as dependable predictors and autonomous risk factors for intermittent positive pressure ventilation [14]. However, an investigation of these particular cytokines is rarely conducted in routine healthcare settings. Hence, if a routine hematological profile can assist in predicting the severity of COVID-19 infection, it may offer additional benefits. Prior research has established a correlation between acute influenza A infection and elevated frequencies of intermediate monocytes in the bloodstream of patients [15]. Peripheral monocytosis, characterized by a cell count above 0.8 × 10^9^ cells/L, has been identified in an additional emergency-oriented study as a potential predictor of adverse outcomes in the emergency department [16]. A recent study indicated that an increase in monocytes detected in COVID-19 patients was related to severity due to dynamic changes in monocyte subsets and their role in the immune response. Elevated levels of monocyte activation markers such as sCD14, CRP, sCD163, and sTissue Factor (sTF) indicate increased immunological activity and inflammation, which contribute to illness severity [17]. Thus, our discovery of monocytosis as a major component in predicting pneumonia could be linked to an inflammatory process. For the next pandemic, we may employ laboratory tests to categorize the degree of illness, such as monocytosis, to assist in anticipating case severity and prioritizing treatment.

In our study, favipiravir elicited mild adverse effects, including nausea, a rash, and bluish corneal discoloration; no further symptoms were reported. Bluish corneal discoloration may occur as a side effect of favipiravir; however, it typically resolves itself on its own [18]. The adverse effects found were modest. There was one observational study that reported an extended length of stay in children who received favipiravir, but the setting of the study was retrospective and not a well-controlled design, so this conclusion is still unclear [19]. In one trial, children with chronic liver disease with COVID-19 infection were successfully treated with favipiravir and had a favorable outcome without side effects [20].

Our study has certain limitations, firstly due to its retrospective nature, as it was conducted at a single center with a restricted sample size. This hinders the generalizability of the findings to broader populations and limits our ability to draw definitive conclusions regarding the effectiveness of favipiravir treatment and the implications of monocytosis as a risk factor for COVID-19 pneumonia in pediatric patients. Additionally, the study’s focus on a specific timeframe during the COVID-19 pandemic may not fully capture the evolving nature of the disease, especially considering the variability in disease severity and treatment practices across different phases of the pandemic.

## 5. Conclusions

Children hospitalized with COVID-19 typically exhibit mild-to-moderate symptoms, such as a fever and a cough. In the initial phase of the pandemic, the COVID-19 pneumonia rate was fairly high. Monocytosis is a significant factor associated with COVID-19 pneumonia. In our practice, favipiravir has been used as a standard treatment, with only mild adverse effects.

## Figures and Tables

**Table 1:** Demographic data, clinical symptoms, and treatment of hospitalized children with COVID-19 infections.

Variable	Total *N* = 349
*Gender*, *n* (*%*)	
Male	181 (51.9)
Female	168 (48.1)
*Age* (*year*), *median* (*IQR*)	8 (3–14)
*Underlying diseases*, *n* (*%*)	
Chronic lung disease	30 (11.5)
Asthma	6 (1.7)
Allergic rhinitis	4 (1.1)
Congenital cyanotic heart disease	1 (0.3)
*Symptoms*, *n* (*%*)	
Fever	349 (100)
Cough	259 (74.2)
Rash	87 (24.9)
Diarrhea	18 (5.2)
*Invasive mechanical ventilation*, *n* (*%*)	4 (1.6)
*HHHFNC*, *n* (*%*)	7 (2)
*PICU admission*, *n* (*%*)	10 (2.9)
*Death*, *n* (*%*)	1 (0.3)
*Length of stay* (*days*); *median* (*IQR*)	12 (10–13)

HHHFNC; heated humidified high-flow nasal cannula, IQR; interquartile range, PICU; pediatric intensive care unit.

**Table 2:** Laboratory results for hospitalized children with COVID-19 infections.

Variables	Results
CRP-QT (mg/L), median (IQR)	1 (0.49–4.59)
CBC	
Hemoglobin (g/dL), median (IQR)	12.60 (11.60–13.30)
Hematocrit	37.50 (34.90–44.30)
White blood cell (cells/µL)	7940 (5630–9900)
Neutrophil %, median (IQR)	44.50 (28.00–54.20)
Lymphocyte %, median (IQR)	44.00 (35.30–62.00)
Monocyte %, median (IQR)	8.00 (5.80–9.60)
Eosinophil %, median (IQR)	1.20 (0.60–2.70)
Basophil %, median (IQR)	0.20 (0–0.40)
Platelet count (cells/µL), median (IQR)	291,000 (247,000–363,000)

**Table 3:** Factors associated with COVID-19 pneumonia in hospitalized children.

Variables	Pneumonia (*n* = 161)	No Pneumonia (*n* = 158)	Crude Odds Ratio	95% CI	*p*-Value
Gender					0.087
male	107 (56.02)	74 (46.84)	1		
female	84 (43.98)	84 (53.16)	0.69	0.45–1.06	
Age (year), median (IQR)	8 (3–14)	9 (3–14)	0.99	0.95–1.03	0.502
Underlying diseases, *n* (%)	27 (14.14)	14 (8.86)	1.69	0.86–3.35	0.124
CRP (mg/L), (IQR)	1.45 (0.48–6.47)	1 (0.50–1.85)	1.24	1.10–1.40	<0.001
Monocytosis, *n* (%)	85 (58.62)	3 (3.57)	38.25	11.53–126.84	<0.001

CRP: C-reactive protein.

## Data Availability

The datasets generated and/or analyzed during the current study are not publicly available but are available from the corresponding authors (R.U.) upon request.
